# LACK OF POLICIES, TRAINING, AND SPECIAL CARE UNITS FOR OBESITY CARE IN PENNSYLVANIA NURSING HOMES

**DOI:** 10.1093/geroni/igz038.2579

**Published:** 2019-11-08

**Authors:** John A Harris, Nicholas Castle

**Affiliations:** 1 University of Pittsburgh, Pittsburgh, Pennsylvania, United States; 2 WVU, Morgantown, United States

## Abstract

It is unclear how nursing homes in the U.S. prepare for the specific needs of residents with obesity at a population level in terms of equipment availability, policies, staff training, and special care units. Using a mail survey of Directors of Nursing (DON) to 420 Pennsylvania Nursing Homes in 2017 and 2018, we examined the reported presence of obesity-specific equipment availability, organizational policies, staff training, and special care units. We compared the presence of these adaptation approaches by whether the DON strongly agreed that obesity was a problem for resident and staff safety using χ2 tests. One hundred fifty-one surveys were returned and included in the analysis (response rate of 36%). 80.7% of respondents were, on average, very concerned when asked about 11 resident medical, functional, relational, and staff-related safety outcomes (e.g., pressure ulcers, hospital readmissions, social isolation, and staff injury). DONs reported reduced equipment availability in nursing homes for obesity-specific beds (66%), walkers (34%), bedside commodes (30%), and gowns (28%). The presence of obesity-specific organizational policies (44%), staff training (26%), and special care units (7%) was limited. DON strong agreement with obesity-related resident and staff safety issues was significantly associated with obesity-specific bed availability (p=0.04) but was not significantly associated with obesity-specific organizational policies (p=0.17), staff training (p=0.51), and special care units (p=0.09). Despite a high concern for resident and staff safety related to obesity care expressed by DONs, there is little appropriate nursing home organizational response as measured by policies, staff training or special care units.

